# GDF15 predict platinum response during first-line chemotherapy and can act as a complementary diagnostic serum biomarker with CA125 in epithelial ovarian cancer

**DOI:** 10.1186/s12885-018-4246-4

**Published:** 2018-03-27

**Authors:** Dan Zhao, Xiaobing Wang, Wei Zhang

**Affiliations:** 1 Department of Gynecological Oncology, National Cancer Center/Cancer Hospital, Chinese Academy of Medical Sciences and Peking Union Medical College, Beijing, 100021 People’s Republic of China; 2 State Key Lab of Molecular Oncology, Laboratory of Cell and Molecular Biology, National Cancer Center/Cancer Hospital, Chinese Academy of Medical Sciences and Peking Union Medical College, Research Building, No.17 Panjiayuan Nanli, Chaoyang District, Beijing, 100021 China; 3 Tumor Marker Research Center, National Cancer Center/Cancer Hospital, Chinese Academy of Medical Sciences and Peking Union Medical College, Research Building, No.17 Panjiayuan Nanli, Chaoyang District, Beijing, 100021 China

**Keywords:** GDF15, CA125, Epithelial ovarian carcinoma, Diagnosis, Chemotherapy

## Abstract

**Background:**

Growth differentiation factor 15 (GDF15) has attracted much interest as a novel biomarker for epithelial ovarian carcinoma (EOC). Research focus has been directed at GDF15 as a diagnostic detection, while the prognostic determination of GDF15 in EOC patients remains to be clearly elucidated. The present study aimed to investigate GDF15 level relative to clinicopathological characters, chemoresponse, and clinical outcome of EOC patients.

**Methods:**

Serum from 122 patients with primary diagnosed EOC were analyzed for GDF15 and serum cancer antigen 125 (CA125). All cases were treated with debulking surgery and first-line chemotherapy, and samples were obtained just before debulking surgical treatment and first-line chemotherapy. Subsequently, clinical characteristics, responses to chemotherapy and progression-free survival (PFS) were recorded.

**Results:**

Increasing levels of serum GDF15 was significantly associated with FIGO stage and lymphonodus metastasis. GDF15 and CA125 detection are complementary in the diagnosis of EOC and can be simultaneously profiled. The chemo-resistant EOC patients (median, 1225.0 pg/mL) showed significantly higher GDF15 than chemo-sensitive patients (median, 824.2 pg/mL; *P* = 0.013). Highly expressed GDF15 was an independent negative prognostic indicator in the PFS (*P* = 0.026) of the 122 EOC cases in the multivariate analysis. Additionally, patients with high level of serum CA125 significantly associated with suboptimal (*P* = 0.043) debulking surgery.

**Conclusions:**

Our results provide valuable evidence that GDF15 is related with first-line chemo-resistance, with highly expressed GDF15 being a strong and an independent indicator of shorter PFS in EOC patients.

**Electronic supplementary material:**

The online version of this article (10.1186/s12885-018-4246-4) contains supplementary material, which is available to authorized users.

## Background

Epithelial ovarian cancer (EOC) is a common gynecological malignant tumor which seriously threatens women’s health [1]. Despite high remission rates following extensive surgical resection of all visible tumors and subsequent postoperative adjuvant carboplatin and paclitaxel chemotherapy, the survival for patients with advanced EOC is still pessimistic because the disease-free interval is short and often transient [2]. The primary obstacle in treatment of EOC remains as resistance to platinum-based chemotherapy [3, 4]. Consequently, with the perspective of personalized treatment, there is an apparent need to introduce novel, strategic tools for the selection of patients. The prediction of patient with EOC who is resistant to first-line chemotherapy can allow making the correct selection of drugs that function via other mechanisms, and discovery of novel therapeutic strategies.

Serum cancer antigen 125 (CA125) is well known and has been used for a long time, representing the “gold standard” biomarker for EOC [5, 6], and has played an important role in the clinical diagnosis, optimal surgery indication and chemotherapy response assessment of patients with EOC. However, there are some limitations, such as elevated CA125 which is only found in about 50% of stage I-II patients with EOC. Further, a consensus has not yet been reached in predicting chemotherapy ​​sensitivity and survival by preoperative serum CA125 level in patients with EOC [7–10]. As the tumor biomarkers for predicting chemo-resistance remains limited, the determination of novel biomarkers will be beneficial in strengthening surveillance of the disease and make reasonable clinical decisions, presenting the best opportunity for successful treatment and improved outcome.

Growth differentiation factor 15 (GDF15), which is also commonly called macrophage inhibitory cytokine-1 (MIC-1), is a secreted cytokine of TGF-β superfamily [11–13]. Numerous studies have shown that serum GDF15 was significantly increased and correlated with clinical stage, lymphonodus involvement, and poor prognosis in a variety of epithelial malignancies, such as, esophageal squamous cell carcinoma, colorectal cancer, non-small cell lung cancer and gynecological malignancy [14–27]. Furthermore, several studies have implied that elevated presurgerical serum GDF15 may be valuable, serving as an indicator in assessment of the treatment response [24, 27]. However, the potential roles of serum GDF15 as a candidate predictor of chemo-resistance and clinical outcome have not yet been investigated comprehensively. Thus in our present research, we attempted to identify the relationship of presurgerical serum GDF15 with clinicopathological characters, as well as, the response to first-line chemotherapy and progression-free survival (PFS) outcomes in patients with EOC, and compare the results with those of CA125 level measurements.

## Methods

### Study population

A retrospective research was performed on patients pathologically diagnosed with EOC and healthy subjects (by physical examination) between January 2009 and April 2013 in our hospital (National Cancer Center/Cancer Hospital, Chinese Academy of Medical Sciences; CICAMS). The patients were selected in this study according to following criteria: 1) Patients were primary subjects who had not received earlier neoadjuvant radiotherapy or chemotherapy, that is, the cases were first identified without previous treatment. 2) All of the EOC patients were confirmed histologically and underwent an extensive cytoreductive operation, followed by the same adjuvant platinum-based chemotherapy (paclitaxel 175 mg/m2 i.v. at day1 and carboplatin AUC 5 i.v. at day 2) in accord to the standard dose calculation formula, without other supplementary chemotherapy. 3) Patient with chronic or acute inflammatory diseases was excluded from the study. 4) In addition, the case was ineligible if their histological diagnosis found other conditions upon pathological review. The control healthy subjects were randomly selected from healthy population who visited medical center for physical examination in our hospital, and confirmed by negative results in ultrasound and CT examination. Finally, 122 patients diagnosed with EOC by histopathological analysis and 120 healthy age-matched subjects confirmed by negative results in ultrasound and CT examination were obtained in this study. Lymph node metastasis was assessed by pathological diagnosis. Only suspicious lymph nodes removed for pathological diagnosis if significant enlarged lymph nodes were detected in the preoperative CT images or found in the operation, or lymph node dissection (pelvic and/or para-aortic) was performed for further pathological diagnosis. The clinical characteristics such as histological grade, residual tumor size, surgical Federation of International of Gynecology and Obstetrics (FIGO) stage (FIGO 2014) [28], and laboratory results were retrospectively acquired from medical records, shown in Additional file 1: Table S1. This research has received ethics approval from the Ethics Committee of CICAMS.

### Definition of clinical response

Residual tumor was defined as the maximal dimension of single largest cancer nodule at the end of cytoreductive surgery. The maximal width of residual tumor recorded as less than or equal to1 cm was considered optimal debulking operation; if greater than 1 cm, it was determined as suboptimal debulking operation. Patients received periodical follow-up following the treatment completion. The response and progression to the treatment was determined by the imaging findings and serum CA125. Progression-free survival (PFS) was defined as the duration from the time of diagnosis to first instance of disease progression or confirmed recurrence, or to the last follow-up date of a disease-free status. Chemosensitivity was defined as a time interval of 6 months or longer between the completion of platinum-based chemotherapy and disease progression or the detection of relapse; if lower than 6 months, it was determined as chemo-resistance.

### Sample preparation and the quantification of GDF15 and CA125

Blood samples were obtained and preserved at − 70 °C in our hospital, retrospectively, at two different time points of baseline/before surgery and 4 weeks after surgery/before chemotherapy. Samples were slowly thawed once for analyses. Serum GDF15 was measured with sensitive ELISA developed by CICAMS, having 20 pg/mL detection limit level and less than 10% coefficient of variation, as described previously [24, 27]. All samples were assayed in duplicate. Serial serum CA125 concentrations were obtained from medical record and detected by the use of a chemiluminescent Architect® enzyme immunoassay from Abbott Laboratories (Abbott Park, IL, USA) and related kit. The cut-offs for CA125 was 35 U/L.

### Statistical analysis

Statistical analysis was performed with the SPSS 19.0 (SPSS Inc., Chicago, IL). The concentrations of serum biomarkers were compared for two and multiple groups by the use of the Mann-Whitney and Kruskal-Wallis test where appropriate, and the Wilcoxon test was used to compare paired samples. Descriptive statistic was applied for demographic information and summarized as the mean value with standard deviation (SD) or range, which analyzed by student’s t-Test. Serum GDF15 and CA125 concentrations are reported herein as the median. Categorical variable was evaluated by Chi-squared test method. Receiver operating characteristic (ROC) curves were assessed to identify the diagnostic performance of GDF15 or CA125 and compared by using the DeLong mathematical model; additionally, logistic regression was also fitted to merge diagnostic information of biomarkers. Survival was evaluated by using the Kaplan–Meier curve and log-rank method. Finally, Cox’s proportional hazard model was conducted for the multivariate analysis. Only variable with *P* value ≤ 0.10 in univariate analysis was included in multivariate model. The statistical significance level was set at a two-sided *P* < 0.05.

## Results

### Elevated levels of serum GDF15 and CA125 were noticeable in EOC

**Fig. 1:**
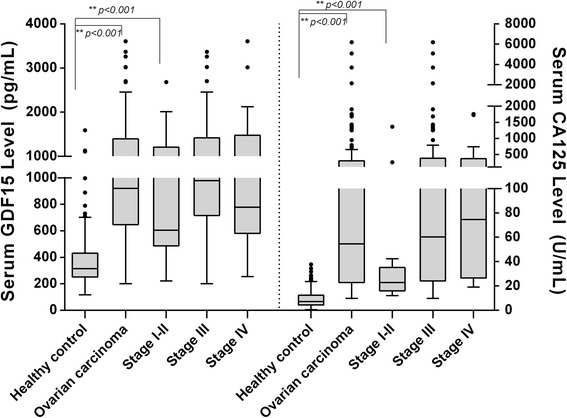
The level of serum GDF15 and CA125 in patients with EOC and healthy control. Serum GDF15 and CA125 in patients with EOC is significantly higher than that of others (*P* < 0.0001), notably, an elevated level of GDF15 was significantly presented in the stage I-II. Moreover, the gradual elevation in serum GDF15 and CA125 was clearly discernible, with significantly higher serum level in stage III -IV. Ovarian carcinoma refers to the group of all patients with ovarian cancer, Stage I-IV refers to the stage I to IV of ovarian cancer patients individually

We detected increased levels of GDF15 (median, 920.9 pg/mL) in the pretreatment serum of EOC patients compared with healthy subjects (median, 286.1 pg/mL, *p* < 0.001; Fig. 1). The serum GDF15 levels varied and gradually increased with FIGO stage, in particular, the level of GDF15 in stage I-II EOC patient (604.6 pg/mL, *p* = 0.018) was significantly increased in comparison with healthy controls, suggesting that the increased serum GDF15 might present in the early stage of EOC. The data also indicated that increased serum GDF15 did not significantly correlate with age and pathological type (Table 1). In like manner, we found that serum CA125 concentration (median, 54.7 U/mL) in EOC patients was distinctly higher than healthy population (median, 7.18 U/mL, *p* < 0.001; Fig. 1), and the difference of serum CA125 level is more significant than the GDF15.

**Table 1:** The association of GDF15 and CA125 with the clinicopathologic parameters in the EOC patients

	n	GDF15(pg/mL)			CA125(U/L)		
		median	IQR^a^	p^b^	median	IQR^a^	p^b^
Age							
≤ 50 y	50	824.7	719.1	0.498	31.8	97.4	0.162
> 50 y	72	990.6	767.6		73.0	399.9	
Histology							
Serous	90	920.9	667.5	0.784	60.4	345.7	0.418
Nonserous	32	923.6	1397.8		35.2	231.8	
Grade							
Low/Medium	41	923.5	715.2	0.496	67.2	448.4	0.122
High	81	912.5	898.8		39.7	99.74	
FIGO							
I/II	13	604.6	719.0	0.088	22.9	19.1	0.005
III/IV	109	943.2	753.5		61.0	348.5	
LN metastasis							
Yes	69	994.0	748.1	0.033	107.3	427.9	0.005
No	53	784.7	770.6		29.1	107.5	

a. IQR: the interquartile range

b. The *p* value was calculated using Mann-Whitney test method

### Serum GDF15 has a better diagnostic performance in EOC, compared with CA125

**Fig. 2:**
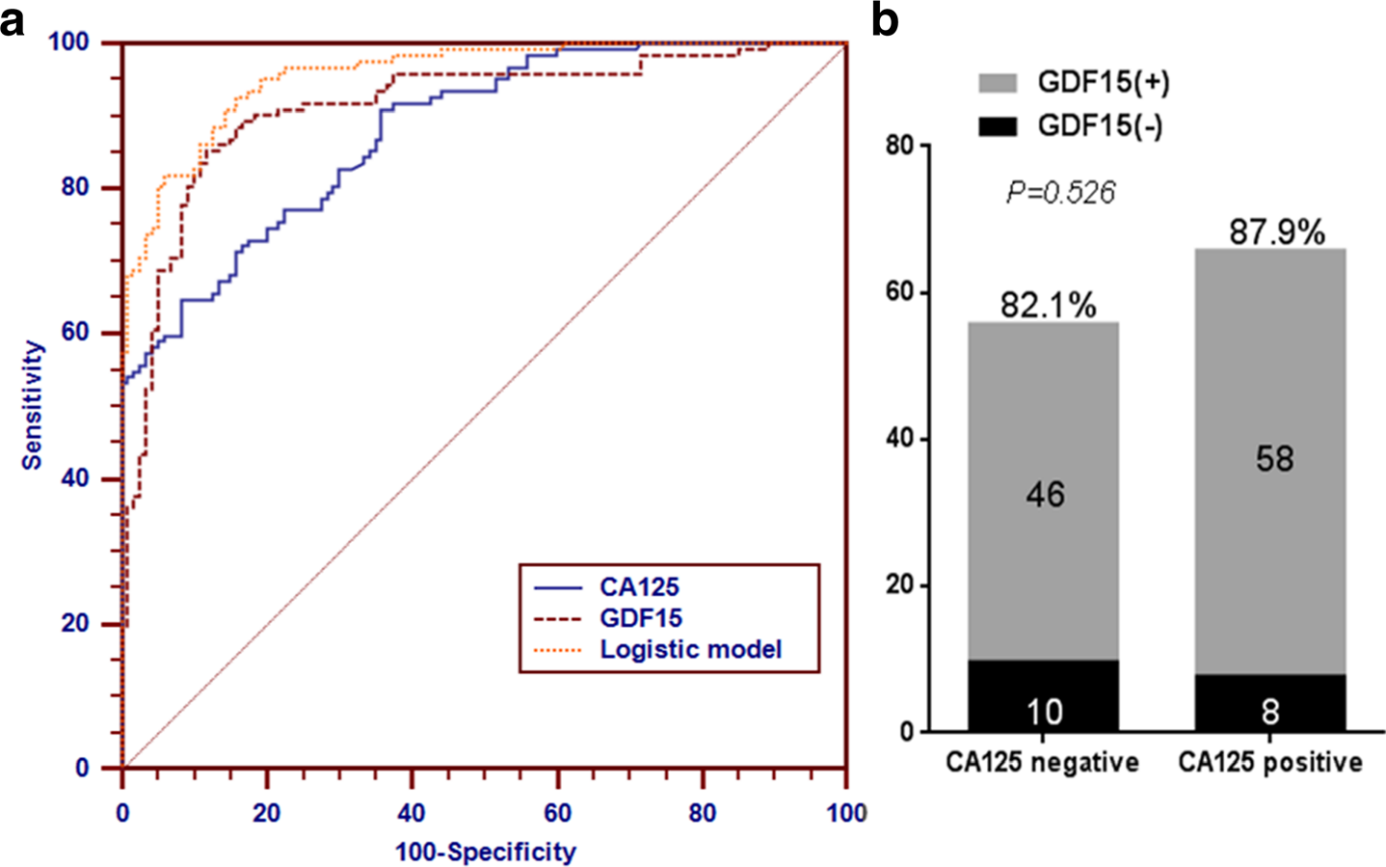
Comparison of the diagnostic performance of serum GDF15 and CA125 for EOC. **a** Sensitivities and specificities of GDF15 and CA125 for the diagnosis of EOC were compared through the analysis of ROC curves. AUROC curve of serum GDF15 was larger than that of CA125 (*P* < 0.033), and the logistic model of GDF15 and CA125 significantly enhanced the efficacy of biomarkers. **b** A similar positive rate (present above the bar) of serum GDF15 (using the cut off value 519.6 pg/mL) was observed in patients with EOC with varying CA125 levels. GDF15+ means the GDF15 level is higher than the median value of the case group; CA125 positive means CA125 level is higher than 35 U/ml

The increased expression of serum GDF15 as a noninvasive biomarker for EOC was evaluated by generating ROC curve and further comparing with CA125. Using the 120 normal samples as negative controls, the calculated area under curve of GDF15 (AUC: 0.913, 95%CI: 0.875–0.951) for EOC is comparable with that of CA125 (AUC: 0.880, 95%CI: 0.840–0.920; *P* = 0.033; Fig. 2a). The Youden’s Index of GDF15 in the diagnosis of ovarian cancer is 0.736 at the 519.6 pg/mL clinical reference value by the ROC curve, and the sensitivity and specificity of GDF15 in diagnosis ovarian cancer were 85.3% and 88.3%. While the diagnostic sensitivity and specificity of CA125 in ovarian cancer were 84.7% and 91.8%, respectively, according to the Youden’s Index. The diagnosis of GDF15 for EOC demonstrated comparable sensitivity and specificity with CA125. Although at the clinical cutoff of 35 U/ml for CA125, the sensitivity will decrease and specificity will increase, the result still indicated that serum GDF15 is a candidate sensitive tumor marker compared to CA125 for the detection of EOC.

### Complementary values of CA125 and GDF15 make them candidates for early detection of EOC

There were significant differences between the two methods by comparing the GDF15 and CA125 diagnostic results (χ2 = 50.933, *p* < 0.001), which indicated that both markers are complementary in the diagnosis of EOC and can be carried out in combination. Serum GDF15 manifested superiority even in those EOC patients with negative CA125 (< 35 U/mL; *n* = 56), showing a median serum GDF15 level of 1291.8 pg/mL and a sensitivity of 82.1% (Fig. 2b). Further study showed that the pathological types of CA125 negative patients were mostly non-serous ovarian adenocarcinoma, indicating GDF15 could complement the application of CA125 in those non-serous pathological types. Logistic regression indicated that the consolidation of GDF15 and CA125 could significantly enhance the diagnostic performance (AUC, 0.957; 95% CI, 0.936–0.979). To further evaluate the performance of GDF15 in early EOC determination, a subgroup comprised of early-stage EOC patients were evaluated (FIGO stage I/II; *n* = 13). The serum GDF15 showed a superior performance (AUC: 0.849, 95%CI: 0.718–0.980) compared with CA125 (AUC: 0.774, 95%CI: 0.646–0.902) in distinguishing early-stage EOC from healthy populations by the ROC analysis. Notably, GDF15 alone could achieve 76.9% (10/13) positive diagnosis rate in early-stage EOC patients (FIGO stage I/II) whereas CA125 could only detect 23.1% early-stage patients, indicating that GDF15 could be applied as a potential candidate for early-stage EOC detection.

### The role of serum GDF15 levels in monitoring surgical treatment of EOC

Wilcoxon analysis showed the median levels of serum GDF15 were only mildly increased from 920.9 pg/mL pg/mL to 946.6 pg/mL (*p* = 0.473) at 4 weeks after surgical resection, while median serum CA125 levels were decreased from 54.7 U/mL to 29.2 U/mL (*p* < 0.001), indicating that CA125 is superior to the GDF15 for the assessment of the tumor burden. The results also suggested that preoperative CA125 level in optimal debulking group was significantly lower than that of suboptimal debulking group (*p* = 0.043), suggesting that preoperative CA125 levels significantly correlated with the size of residual tumor, thus can be used to evaluate the effect of cytoreductive surgery, while GDF15 has no such clinical value (Table 2). Further analysis of the paired serum before treatment and a month after surgery of the patients with EOC, shown that serum CA125 concentration in 99 patients was decreased; while the reduction of the GDF15 level was observed in only 67 cases. We found that CA125 level was slightly elevated in 23 patients following surgery, and pathological type was mostly non-serous ovarian adenocarcinoma; while 55 patients with increasing level of GDF15 have no association with such pathological types. The result suggested that GDF15 and CA125 can be used as mutually reinforcing indicators, although GDF15 was significantly inferior to CA125 in monitoring the debulking surgical treatment.

**Table 2:** The association of clinical character with preoperative serum levels of CA125 and GDF15 levels in 122 EOC patients

Patient number	Total population (*n* = 122)	Debulking surgery		*P*-value		Chemotherapeutic response		*P*-value
		optimal(*n* = 84)	Suboptimal (*n* = 38)			Chemosensitive(*n* = 91)	Chemoresistant(*n* = 31)	
Age (mean ± s.d.)	53.4 ± 11.1	52.6 ± 11.6	55.1 ± 9.7	0.251^a^		52.4 ± 11.2	54.8 ± 10.9	0.302^a^
Histology								
Serous	90 (73.8%)	61	29	0.836^b^		66	24	0.765^b^
Non-serous	32 (26.2%)	23	9			25	7	
FIGO								
Stage I/ II	13 (10.7%)	11	2	0.341^b^		13	0	0.037^b^
Stage III/IV	109 (89.3%)	73	36			78	31	
Grading								
Low/Medium	41 (58.2%)	29	12	0.911^b^		32	9	0.686^b^
High	81 (41.8%)	55	26			59	22	
Debulking surgery								
Optimal	84 (68.9%)					62	22	0.944^b^
Suboptimal	38 (31.1%)					29	9	
LN metastasis								
Yes	69 (56.6%)	43	26	0.114^b^		48	21	0.213^b^
No	53 (43.4%)	41	12			43	10	
CA125 (median,U/L)	54.7	34.4	106.8	0.043^c^		33.1	74.6	0.108 ^c^
GDF15 (median,pg/mL)	920.9	933.3	915.4	0.596^c^		824.2	1225.0	0.013 ^c^

a. The *p* value was calculated using t-test; b. The *p* value was calculated using Chi-squared test; c. The *p* value was calculated using Mann-Whitney test

### High pretreatment serum GDF15 levels significantly associated with chemotherapy sensitivity of patients with EOC

The features of clinicopathological characters predicting chemo-resistance are indicated in Table 2. The results revealed that GDF15 level in the sensitive patients’ group was lower than the resistance group significantly (*p* = 0.013); while no such significant difference (*p* = 0.108) was observed for pretreatment CA125 levels. In this research, 31 out of the 122 patients demonstrated chemo-resistance; the overall chemo-resistance rate was 25.4%, with 26.9% chemoresistance rate in positive GDF15 group compared with 16.7% in negative GDF15 group (*P* < 0.001), indicating that pretreatment GDF15 levels significantly correlated with the occurrence of drug resistance in patients with EOC and may be used as predictors of drug resistance. In addition, FIGO stage was also significantly associated with the chemotherapeutic response.

### Elevated pretreatment serum GDF15 negatively associated with prognosis of patients with EOC

**Fig. 3:**
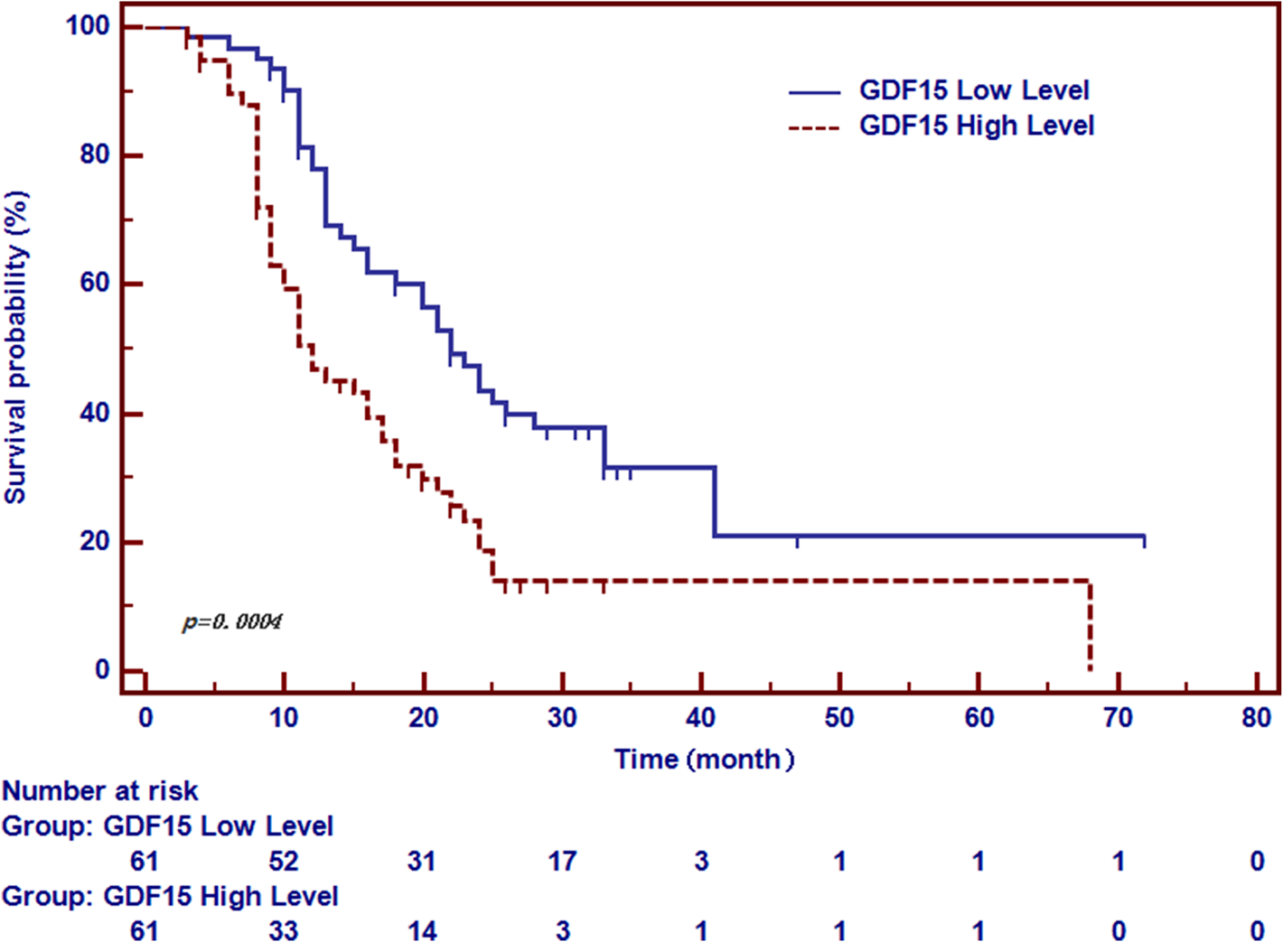
The value of serum GDF15 in the prediction of progression-free survival of patients with EOC. Progression-free survival (PFS) were prepared and analyzed between two divided groups according to the median levels of serum GDF15 in patients before treatment. Patients with higher serum GDF15 demonstrated a trend for poorer PFS (*P* = 0.0004)

PFS duration is one of the most crucial clinical events associated with unfavorable prognosis of EOC before death. With a median follow-up duration of 18 months (range 3–72), clinicopathological characters for prediction of PFS are shown in Table 3. According to the calculated median value of serum GDF15 and CA125, Patients were classified into presurgerical low-level group and presurgerical high-level group. A log-rank analysis indicated that EOC patients with higher serum GDF15 (*P* = 0.0004) exhibited a trend of poorer progressive-free survival (PFS), revealed by the analysis of the follow-up data (Fig. 3 and Table 3), while pre-operative CA125 values did not show appreciable statistical significance. Additionally, FIGO stage (*P* = 0.0001) and residual tumor size (*P* = 0.016) were significantly associated with PFS duration by the univariate analysis. However, multivariate analysis demonstrated that only clinical FIGO stage (HR: 3.521, 95%CI: 1.044–11.880; *p* = 0.044) and GDF15 (HR: 1.660, 95%CI: 1.065–2.586; *p* = 0.026) were independent prognostic characters associated with unfavorable PFS.

**Table 3:** Univariate and Multivariate Cox regression analysis for PFS of patients with EOC

Clinicopathologic characteristics	Univariate		P	Multivariate		P
	HR	95% CI		HR	95% CI	
Age, y						
≤ 50 vs > 50	1.0268	0.668–1.578	0.901			
FIGO stage						
I/II vs III/IV	6.528	3.808–11.190	0.0001	3.521	1.044–11.880	0.044
Tumor grade						
Low/Medium vs High	0.971	0.632–1.491	0.888			
Histology						
Serous vs Nonserous	0.616	0.385–0.984	0.062	0.626	0.354–1.105	0.108
Residual tumor, cm						
≤ 1 vs > 1	1.670	1.032–2.703	0.016	1.380	0.872–2.185	0.171
CA125						
Low vs High	1.327	0.864–2.037	0.176			
GDF15						
Low vs High	2.057	1.326–3.190	0.0004	1.660	1.065–2.586	0.026

HR: hazard ratio; CI: confidence interval

Low/high group means lower or higher than the calculated median value of presurgerical serum GDF15 and CA125, respectively

## Discussion

The identification of non-invasive biomarkers for predicting the response of EOC patients to chemotherapy will improve prognosis and represent an important direction for reduction of mortality resulting from EOC [29, 30]. To date, CA125 is the most established serologic marker in advanced ovarian cancer [5, 8, 31]. Therefore, in our present study, CA125 was used as a benchmark biomarker. Additionally, we evaluated GDF15 as a serum tumor biomarker regarding the detection of EOC, especially, as a prognostic marker for prediction of chemotherapy resistance. To our present knowledge, our research stands as the first to investigate potential clinical performance of serum GDF15 in EOC patients in both diagnosis and during post-operative chemotherapy, and its capacity is complemented when offered to CA125 negative individuals and early-stage patients.

First, we found that patients with EOC have a much higher serum GDF15 compared with normal controls as previously reported [23], suggesting that serum GDF15 could be applied as a potential seromarker for differential detection of EOC. Previously study also indicated that median plasma GDF-15 concentration was elevated in ovarian cancer as compared to women with benign ovarian tumors (*p* < 0.001); additionally, GDF-15 plasma concentration correlated inversely with survival time and was an independent predictor of survival, after correction for FIGO stage and age (*p* = 0.01). In this study, we also found that serum GDF15 in early-stage EOC patients (FIGO stage I/II) were dramatically higher than healthy subjects, and the serum GDF15 remained elevated with the clinical stage of the tumor, compared to controls. These results indicate that highly elevated levels of serum GDF15 might have occurred at the early stage and corresponded to the progression of EOC. In addition to ovarian cancer, a study reported that high plasma GDF15 was significantly associated with FIGO stage III/IV disease and lymph node metastases (*p* < 0.001) in large validation cohort of endometrial carcinomas, further suggested that GDF15 may be significantly associated with lymph node metastasis, possibly applicable for all gynecological tumors which worth further exploration [32]. Nevertheless, the consequence or biological roles of serum GDF15 elevation in EOC remain to be elucidated [33]. Until now, the research hotspots have been directed to explore the strength of GDF15 as a potential diagnostic molecular biomarker for EOC. A few publications have demonstrated the performance of serum GDF15 in distinguishing malignant and benign ovarian tumors [24, 34–36]. In our research, GDF15 indicated high sensitivity of 85.3% at 88.3% specificity, consistent with reports from previous studies regarding serum GDF15 in EOC, with slightly differences in detective sensitivity, possibly associated to patient characteristics [23]. Moreover, we demonstrate parallel diagnostic specificity and sensitivity. Compared with CA125, GDF15 showed a higher diagnostic sensitivity in early-stage EOC; while the median preoperative serum GDF15 level of the 13 patients with stage I-II EOC was only 604.6 pg/mL, with a range of 222.1–2681.8 pg/mL. This reveals a possible limitation in early detection of the disease because, at these stages, there are fluctuated values in low-level positivity, making it difficult to determine significance.

In our present study, we also found that CA125 and GDF15 levels were mutually complementary and their composite determination could significantly enhance the sensitivity which obtained from individual biomarker alone. The panel with the GDF15 and CA125 (Fig. 2a) acquired by the model of logistic regression demonstrated better diagnostic accuracy (AUC = 0.957) in differencing EOC from normal population. GDF15 improves the utilization of CA125 as a serum biomarker in EOC, and simultaneously using these two biomarkers enhances the sensitivity in EOC. Therefore, it is favorable to combine GDF15 with clinical proven biomarker CA125 to discriminate EOC from normal with high accuracy. We conclude that GDF15 is a promising, noninvasive seromarker and may be a valuable supplement to serum biomarkers available in use.

Monitoring optimal debulking would facilitate a more precise prognostic stratification, and contribute in improving the efficacy of multimodal therapy [37, 38]. Our results revealed that presurgerical elevated GDF15 level was related to clinical advanced FIGO stages and lymphonodus metastasis in EOC patients. In present study, the serum level of GDF15 and CA125 in patients with EOC was detected and compared before and after surgical treatment. We found that CA125 serum concentrations were significantly lower following cytoreductive therapy, while GDF15 did not show this pattern, suggesting that CA125 is better than GDF15, relative to in-vivo tumor load in ovarian cancer, that is, CA125 levels were significantly associated with postoperative residual tumor size. These give us warnings that patients with elevated serum GDF15 levels may have the possibility of metastases, while CA125 predict worse debulking outcome, which may help gynecologist to determine eligible surgical patients.

It is well-established that biomarker used to predict clinical chemotherapeutic response has great significance in helping EOC patient’s management [29, 30]. Adjuvant chemotherapy has improved PFS in various malignancies [39, 40]. Currently, there is no efficient method for predetecting the clinical response of EOC cases to chemotherapy. Therefore, all EOC patients are customarily treated with first-line chemotherapy without regard for the adverse events, which do not represent a rational and reasonable approach [41]. It would be favorable to hold an advance opportunity to better tailor individual treatment to EOC patient, especially, those with resistance to platinum, and it would be a milestone for successful treatment of women with EOC. In this study, we found that presurgerical serum GDF15 levels significantly correlated with chemosensitivity of EOC, that is, a high level of GDF15 was an indication that there is a high risk of resistance, which could not be discovered in the previous study [23]. The prediction of chemosensitivity before treatment could help the clinician select the appropriate chemotherapy to customize individualized treatment strategy, which can cast light on the failures and successes of treatment in EOC cases and also supply an important basis for individualized therapy, reckoning the primary aim of our study. In addition, the result from follow-up data showed GDF15 levels in patients negatively correlated with PFS (*P* = 0.0004). Multivariate Cox’s analysis indicated that serum GDF15 is an independent predictor for EOC (*P* = 0.026). We will continue to expand and extend the period for obtaining follow-up samples, to explore the relationship between GDF15 and prognosis thoroughly.

There are several limitations concerning our present study. Firstly, we did not include the serum GDF15 level data during or after chemotherapy, hence, we were unable to analyze their connection with progression and remission of the disease. Secondly, this study is limited in its retrospective feature and the fact that it was not possible to obtain samples in strictly consecutive patients, which might have led to a potential selection bias. Therefore, further prospective research is needed to achieve the association between serial detections of GDF15 during or following chemotherapy and sound clinical outcomes in EOC patients. Notwithstanding its limitations, high numbers of patients with optimal surgical debulking and the thorough clinical staging followed by platinum-based chemotherapy was included in this mono-institutional retrospective research, which could be considered as strengths of our study. Additionally, present study involved homogeneous patients who underwent similar extensive surgical procedures and consistent platinum-based chemotherapy regimens, thus could heighten the generalizability of the findings.

## Conclusions

The current research provides further comprehension on the clinical performance of GDF15 by proving that GDF15 is mutual complementary with CA125 and higher GDF15 are involved with chemo-resistance in EOC patients and patients with shorter PFS duration. The association of GDF15 with response to chemotherapy indicates an issue of both academic and clinical interest that may be useful in tailoring chemotherapy in the future. Multi-institutional research will be needed to verify whether GDF15 is really an independent indicator for first-line chemotherapy in patients with EOC.

## Additional file

Additional file 1: **Table S1**. Characteristics of subjects with EOC and controls (DOCX 16 kb)

## Abbreviations

CA125: Serum cancer antigen 125
EOC: Epithelial ovarian cancer
GDF15: Growth differentiation factor 15
MIC-1: Macrophage inhibitory cytokine-1
PFS: Progression-free survival
FIGO: Federation of International of Gynecology and Obstetrics
SD: Standard deviation
ROC: Receiver operating characteristic
