# Supplementary material for: GDF15 predict platinum response during first-line chemotherapy and can act as a complementary diagnostic serum biomarker with CA125 in epithelial ovarian cancer
Source: BMC Cancer. 2018 Mar 27;18:328. doi: 10.1186/s12885-018-4246-4 (PMC5870062; doi:10.1186/s12885-018-4246-4)
Supplement: Supplementary file 1 — Table S1. Characteristics of subjects with EOC and controls (DOCX 16 kb) [file 12885_2018_4246_MOESM1_ESM.docx]

**Table 1. Characteristics of subjects with EOC and controls**

| **Clinicopathological Parameters** | **EOC cases**  **(n=122)** | **Healthy controls**  **(n=120)** |
| --- | --- | --- |
| Age, median (range), y | 55(18-80) | 53(20-75) |
| Baseline CA125, median (range) (U/ml) | 45.0(4.9-6655) | 7.1(4.4-37.8) |
| FIGO stage, n (%) |  |  |
| Ⅰ | 6 (4.9%) |  |
| Ⅱ | 7 (5.7%) |  |
| Ⅲ | 104(85.2%) |  |
| Ⅳ | 5 (4.1%) |  |
| Histology, n (%) |  |  |
| Serous | 90 (73.8%) |  |
| Non-serous | 32 (26.2%) |  |
| endometrioid | 7 (5.7%) |  |
| Clear cell | 10 (8.2%) |  |
| Mucinous | 4 (3.3%) |  |
| Carcinomas (mixed, carcinosarcoma,or undifferentiated) | 11 (9.0%) |  |
| Grading, n (%) |  |  |
| Low | 16 (13.1%) |  |
| Medium | 25 (20.5%) |  |
| High | 81 (66.4%) |  |
| Surgical residual tumor ^a^ |  |  |
| Optimal | 84 |  |
| Suboptimal | 38 |  |
| Lymph node metastasis |  |  |
| Yes | 69 |  |
| No | 53 |  |
| Platinum resistance ^b^ |  |  |
| Yes | 31 |  |
| No | 91 |  |

a.Optimal surgical residual tumor: The maximal width of residual tumor recorded as less than or equal to1 cm; Suboptimal surgical residual tumor: The maximal width of residual tumor recorded as greater than 1 cm.

b. Platinum resistance: Time interval of lower than 6 months between the completion of platinum based chemotherapy and disease progression or the detection of relapse
